# Exposure-Response Modeling to Support Dosing Selection for Phase IIb Development of Kukoamine B in Sepsis Patients

**DOI:** 10.3389/fphar.2021.645130

**Published:** 2021-04-19

**Authors:** Huanhuan Wang, Xiaoyun Hu, Teng Wang, Cheng Cui, Ji Jiang, Kai Dong, Shuai Chen, Chunyan Jin, Qian Zhao, Bin Du, Pei Hu

**Affiliations:** ^1^ Clinical Pharmacology Research Center and State Key Laboratory of Complex Severe and Rare Diseases, Peking Union Medical College Hospital, Chinese Academy of Medical Sciences and Peking Union Medical College, Beijing, China; ^2^ Key Laboratory of Clinical PK and PD Investigation for Innovative Drugs, Beijing, China; ^3^ NMPA Key Laboratory for Clinical Research and Evaluation on Drugs, Beijing, China; ^4^ Medical ICU,Peking Union Medical College Hospital, Union Medical College and Chinese Academy of Medical Sciences, Beijing, China; ^5^ Clinical Research Center for Innovative Drugs, Tianjin Chasesun Pharmaceutical Co., Ltd., Tianjin, China

**Keywords:** SOFA score, sepsis, exposure-response modeling, model validation, simulation

## Abstract

**Aim:** Kukoamine B, a small molecule compound, is being developed for the treatment of sepsis in a Phase II clinical trial. The objective of this study was to optimize dosing selection for a Phase IIb clinical trial using an exposure-response model.

**Methods:** Data of 34 sepsis patients from a Phase IIa clinical trial were used in the model: 10 sepsis patients from the placebo group and a total of 24 sepsis patients from the 0.06 mg/kg, 0.12 mg/kg, and 0.24 mg/kg drug groups. Exposure-response relationship was constructed to model the impact of the standard care therapy and area under curve (AUC) of kukoamine B to the disease biomarker (SOFA score). The model was evaluated by goodness of fit and visual predictive check. The simulation was performed 1,000 times based on the built model.

**Results:** The data of the placebo and the drug groups were pooled and modeled by a nonlinear mixed-effect modeling approach in sepsis. A latent-variable approach in conjunction with an inhibitory indirect response model was used to link the standard care therapy effect and drug exposure to SOFA score. The maximum fraction of the standard care therapy was estimated to 0.792. The eliminate rate constant of the SOFA score was 0.263/day for the standard care therapy. The production rate of SOFA score (K_in_) was estimated at 0.0569/day and the AUC at half the maximal drug effect (EAUC_50_) was estimated at 1,320 h*ng/mL. Model evaluation showed that the built model could well describe the observed SOFA score. Model-based simulations showed that the SOFA score on day 7 decreased to a plateau when AUC increased to 1,500 h*ng/mL.

**Conclusion:** We built an exposure-response model characterizing the pharmacological effect of kukoamine B from the standard care therapy in sepsis patients. A dose regimen of 0.24 mg/kg was finally recommended for the Phase IIb clinical trial of kukoamine B based on modeling and simulation results.

## Introduction

Sepsis is an infection-initiated system inflammatory syndrome. More than 25–30% of patients with sepsis die from the condition, and hospital mortality for septic shock approaches 40–60% ( Annane et al., 2005; Lever and Mackenzie, 2007; Martin, 2012; Singer et al., 2016). Pathogen associated molecule patterns (PAMPs) from infection source can activate the pattern-recognition receptors of the innate immune system, mediate inflammatory cell activation, and release inflammatory factors (Martin, 2012). Current interventions are focused on organ support, infection source control, and antibiotic therapy (Cecconi et al., 2018). The disease severity of sepsis was correlated with the degree of organ dysfunction.

The Sequential Organ Failure Assessment (SOFA) score, used to codify the degree of organ dysfunction, is a simple and effective method to monitor patient condition and disease progression, and is widely used in ICUs to evaluate the severity and prognosis of patients with sepsis (Ferreira et al., 2001; Tan et al., 2018; Lambden et al., 2019). SOFA score is a total score including six domains: respiratory system (PaO_2_/FiO_2_), renal system (creatinine), central nervous system (Glasgow Coma Score), coagulation system (platelet counts), hepatic (bilirubin), and cardiovascular system (systolic blood pressure). Sepsis is now defined in the presence of an infection combined with an acute change of two or more points of the SOFA score (Raith et al., 2017). SOFA values range from 0–25, with higher values denoting higher disease activity. The European Medicines Agency has accepted that a change in the SOFA score is an acceptable surrogate marker of efficacy in exploratory trials of novel therapeutic agents in sepsis (European Medicine Agency, 2006). Thus, SOFA score can be used as the biomarker of pharmacological effect in sepsis patients along with PK exposure to guide the development of novel drugs in clinical trial. To date, no exposure-response models using the SOFA score have been reported.

Lipopolysaccharides (LPS) and oligodeoxynucleotides containing CpG motifs (CpG DNA) are important drug targets for sepsis treatment. Kukoamine B, a major bioactive component of Cortex Lycii (Liu et al., 2011a; Liu et al., 2011b), has high affinities for LPS and unmethylated CpG DNA, and is a developed novel drug that targets both LPS and CpG DNA in the treatment for sepsis (Krieg, 2003; Liu et al., 2009). Kukoamine B was approved for investigation in a Phase I clinical trial in healthy volunteers by the National Medical Products Administration (ClinicalTrials.gov # NCT02219971) in 2014. Pharmacokinetics (PK), pharmacodynamics (PD), and the safety profile of kukoamine B in sepsis patients were investigated in a placebo-controlled, randomized, and double-blind Phase IIa trial, in which SOFA score was evaluated as the biomarker of pharmacological effect (ClinicalTrials.gov #NCT03237728). The decreasing trend of SOFA score across time was preliminarily observed to depend on the dose of kukoamine B. No obvious drug-related adverse effects were found in the drug groups. To recommend a safe and efficient dose for the following Phase IIb study design, exposure-response relationship was established based on Phase IIa clinical trial data.

The objective of our study was to i) develop an exposure-response model using a population approach; ii) differentiate the drug effect from the standard care therapy; iii) simulate the profiles of day 7 SOFA score vs. area under curve (AUC) and recommend an optimized dose for the Phase IIb clinical trial.

## Methods

### Data Source

A multiple-dose, randomized, double-blind, placebo-controlled phase IIa trial (ClinicalTrials.gov #NCT03237728) was conducted in Chinese sepsis patients. In the pre-trial, a total of eight patients were randomized at a 3:1 ratio to the 0.06 mg/kg drug group and the placebo group. In the formal trial, a total of 36 patients were randomized at a 1:1:1 ratio to the 0.12 mg/kg, 0.24 mg/kg, and placebo group. In the placebo group, enrolled sepsis patients received the standard care therapy required by the sepsis bundle such as antibiotics and vasopressor, etc. (Levy et al., 2018). In the drug groups, all enrolled subjects were administrated for 1 h with an intravenous infusion of kukoamine B every 8 h for seven days besides the standard care therapy by the sepsis bundle. The dose of kukoamine B was calculated according to the patient’s bodyweight. SOFA score was evaluated prior to the dose and each day for eight days following dosing. In the pre-trial, blood samples were collected pre-dose and 0.5, 1, 2, 4, 6, and 8 h after the first starting dose, before the last three doses, and 0.5, 1, 2, 4, 6, 8, 12, 16, and 24 h after the last dose. In the formal trial, blood samples were collected pre-dose and 0.5, 1, 2, 6, and 8 h after the first starting dose, before the last three doses, and 0.5, 1, 2, 6, and 12 h after the last dose. The study was conducted in accordance with the ethical principles stated by the Declaration of Helsinki, International Conference on Harmonization Technical Requirements for the Registration of Pharmaceuticals for Human Use of Good Clinical Practice. All participants or their guardians were required to sign a subject information agreement and the protocols of the study were approved by the Peking Union Medical College Hospital (PUMCH) Investigational Review Board (IRB).

### Data Analysis

Kukoamine B concentrations in the plasma of sepsis patients were analyzed using a validated ultra-performance liquid chromatography tandem mass spectrometry (UPLC-MS/MS) method with a limit of quantification (LLOQ) of 0.1 ng/ml (Wang et al., 2017 ). Concentrations vs. time profiles per group were plotted by Phoenix WinNonlin (version 7.0, Certara, NJ, United States). Area under curve (AUC_0–8 h_) of the first dose and the last dose of 19 patients were calculated by non-compartment analysis (NCA) using Phoenix WinNonlin (version 7.0, Certara, NJ, United States), respectively. AUC_0–8 h_ of the first dose of five patients were calculated by NCA because of the missing day 7 PK samples. The exposure-response model was built by a nonlinear mixed-effect modeling approach using NONMEM (version 7.3; Icon Inc., North Wales, PA, United States) in conjunction with Pirana (version 2.8.0, Pirana Software and Consulting BV, Amesterdam, The Netherlands) and the PSN (version 1.1.453) toolkit. Post processing of the NONMEM output was undertaken with Xpose (Version 4.0 preview, release 6), programmed in the statistics package R (version 3.5.1). The FOCEI algorithm was employed in the model-building process. The baseline SOFA as a variable was directly included in the model. The structure of the model was evaluated based on objective function value (OFV), goodness-of-fit plots, the precision of parameter estimates, prediction-corrected visual predictive check (pcVPC, Bergstrand et al., 2011), physiological/pathological plausibility, and shrinkage (Savic et al., 2009). Conditional weighted residuals were calculated.

### The Exposure-Response Model

The data of the placebo and drug groups were pooled for modeling. Pharmacological effect in the drug groups was affected by two factors: i) pharmacological effect of the standard care therapy (Zhang et al., 2019) and ii) pharmacological effect of kukoamine B. The exposure-response model was modeled in Eq. 3 (Hu, 2014; Hu et al., 2014; Hu and Zhou, 2016):

$$\mathrm{SOFA}\left(t\right)=\mathrm{Base}-F_{\mathrm{placebo}}\times \mathrm{Base}\times \left(1-e^{-kt}\right)-\left(1-F_{\mathrm{placebo}}\right)\times \mathrm{Base}\times \left(1-R\left(t\right)\right)+\varepsilon ,$$ (3)

Where SOFA (t) is time-dependent SOFA, $F_{\mathrm{placebo}}$ (0≤ $F_{\mathrm{placebo}}$ ≤ 1) is the maximum fraction of the standard care therapy. $\left(1-F_{\mathrm{placebo}}\right)$ is the maximum fraction of drug effect. Base indicated the baseline SOFA, $\mathrm{Base}\times \left(1-e^{-kt}\right)$ is the change of SOFA which is productive by the standard care therapy, IIV for k is assumed to be normally distributed and is described by an addition model,   R(t) is a latent variable which is productive by kukoamine B. ε  ∼ N (0, σ^2^) represents the residual unexplained variability, which is described by an additional function.

The pharmacological effect of kukoamine B is assumed to be driven by a latent variable as follows:

R (t) governed by:

$$\frac{dR\left(t\right)}{dt}=k_{\mathrm{in}}\times \left(1-\frac{AUC}{AUC+EAUC_{50}}\right)-k_{\mathrm{out}}\times R\left(t\right),$$ (4)

Where AUC is steady state AUC for kukoamine B: $k_{\mathrm{in}}$, $EAUC_{50}$, and $k_{\mathrm{out}}$ are the parameters of the indirect response (IDR) model. $\;EAUC_{50}\;$ is the steady state AUC of kukoamine B that produces half of the maximum attainable inhibition. $k_{\mathrm{in}}$ is the zero-order production rate of the residual percentage of the SOFA score, $k_{\mathrm{out}}$ is the first-order elimination rate of the residual percentage of the SOFA scores. It is assumed that at the baseline, R (0) = 1, yielding $k_{\mathrm{in}}$ = $k_{\mathrm{out}}$. IIV for $k_{\mathrm{in}}$ and $EAUC_{50}$ is fixed to achieve successful OFV minimization. For the placebo group, R (t) = 1 (AUC = 0).

### Model Evaluation

Goodness-of-fit (GOF) plots were used to evaluated the predictive performance of the exposure-response model, including dependent variable vs. individual prediction (IPRED) or population prediction (PRED), individual weighted residuals (IWRES) vs. PRED, and CWRES vs. time. pcVPC was used for model evaluation (n = 1,000) and stratified by the dose to ensure the models performed adequately across subgroups (U.S. Food and Drug Administration, 2019).

### Simulations

To characterize the profiles of day 7 SOFA vs. AUC, the simulation was performed based on the built exposure-response model. The method was as follows: time in the exposure response model was fixed at 7 (t = 7), day 7 SOFA scores were simulated at the baselines 15, 10, and 5 1,000 times when AUC ranged from 0 ng*h/mL to 5,000 ng*h/mL. The 30th, 50th, and 70th percentiles (prediction intervals) of day 7 SOFA scores were plotted against AUC. The profiles of SOFA vs. time were also simulated, and the plot was shown with different colored shaped areas representing the placebo group and the recommended 0.24 mg/kg group in a panel.

## Results

### Patients and Samples

The data of 10 sepsis patients in the placebo group and 24 sepsis patients in the drug groups contributing 245 measurable SOFA scores were finally included into our model dataset. Ten patients were excluded from the SOFA dataset according to the exclusion criteria or due to their withdrawal from the clinical trials. There were 43 missing PK samples due to subjects withdrawing from clinical trials or because the drug was not successfully administrated. The results are shown in Supplementary Table S1. The PK concentrations of all samples except 0 h were more than LOQ. For 19 patients, we calculated the AUC_0–8 h_ of the first dose and the last dose, and the average of them was used as the exposure measure. Five patients dropped out of the clinical trial, so the PK sample of the last dose was missing, and the AUC_0–8 h_ of the first dose was used. AUC (mean ± SD) for the 0.06 mg/kg group was 295.21 ± 47.16 (mean ± SD) h*ng/ml; AUC (mean ± SD) for the 0.12 mg/kg group was 827.85 ± 334.10 (mean ± SD) h*ng/ml; AUC (mean ± SD) for the 0.24 mg/kg group was 1,482.51 ± 378.34 (mean ± SD) h*ng/ml. Supplementary Figure S1 shows the concentration vs. time profiles per group, Supplementary Figure S2 shows the SOFA score vs. time profiles per group. Baseline SOFA (mean ± SD) for the placebo group was 12.10 ± 4.36 (mean ± SD); baseline SOFA (mean ± SD) for the 0.06 mg/kg group was 9.25 ± 2.22 (mean ± SD); baseline SOFA (mean ± SD) for the 0.12 mg/kg group was 9.56 ± 3.09 (mean ± SD); and baseline SOFA (mean ± SD) for the 0.24 mg/kg group was 10.09 ± 3.86 (mean ± SD).

### Exposure-Response Model

A latent-variable approach in conjunction with an inhibitory indirect response model was used to link the standard care therapy effect and drug exposure to SOFA score. The results of the parameter estimation are summarized in Table 1. The OFV value was 526.34.

**TABLE 1:** Parameter estimation results for the exposure-response model.

Parameter	Estimate	Units	RSE%	IIV (RSE%)	Shrinkage (%)
K_in_ ^a^	0.0569	1/day	53	0 FIXED	—
EAUC_50_ ^c^	1,320	ng/mL^a^h	37	0 FIXED	—
k^b^	0.263	/day	29	30.2 (22)	8
F_placebo_ ^d^	0.792		3		—
ɛ^e^	1.96		9	30.2%	6

^a^ The production rate of SOFA score.

^b^AUC at half the maximal effect.

^c^The amelioration rate constant of SOFA score in the placebo group.

^d^The maximal fraction of the standard care therapy.

^e^Additive error.

### Model Evaluation

**FIGURE 1:**
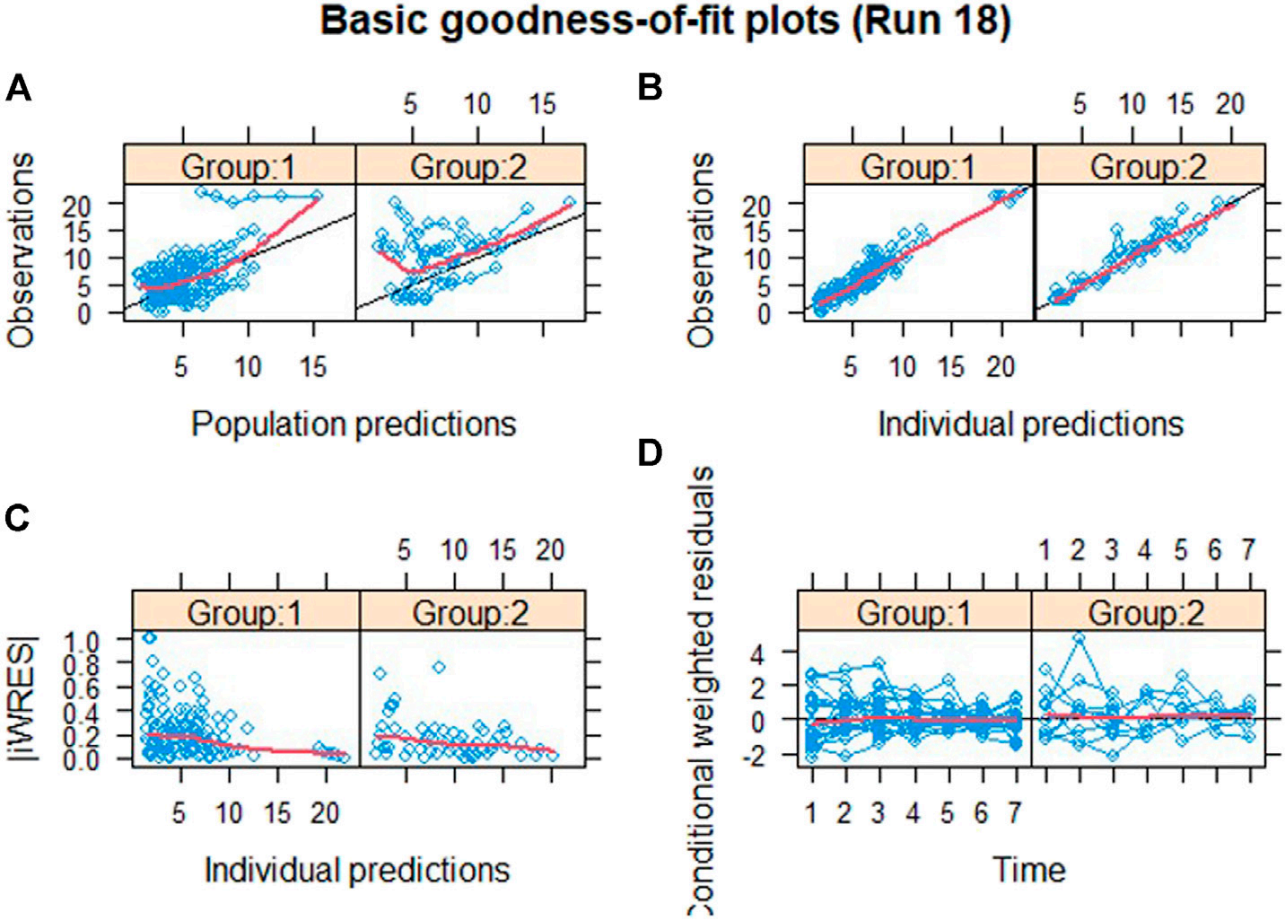
Goodness-of-fit plots. Group 1: the drug group; Group 2: the placebo group; **(A)** population predictions vs. observation. **(B)** Individual predictions vs. observation. **(C)** Individual predictions vs. absolute of individual weighted residuals and **(D)** conditional weighted residuals vs. time. The black solid lines in **(A)** and **(B)** represent the line of identity and that in **(D)** represent the line y = 0. The red solid lines in each panel represent the Loess smooth curve of the data.

**FIGURE 2:**
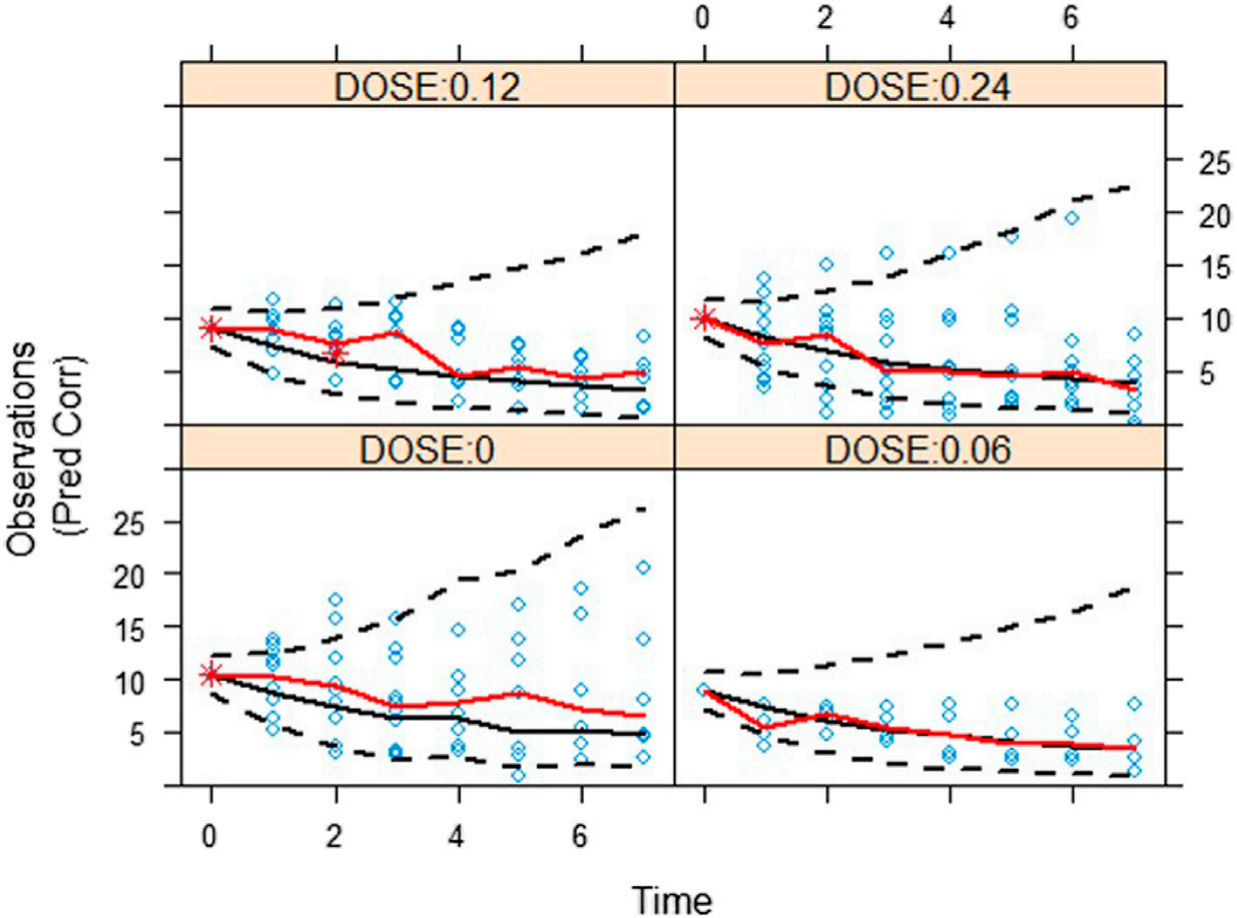
A visual assessment of the prediction-corrected posterior predictive performance (pcVPC) of the drug effect model in terms of its ability to predict the observed SOFA score. Red solid lines are the 50th percentiles for observation. And black dash lines and black solid lines are the 10th, 50th, and 90th percentiles for prediction.

The validity of the exposure-response model was assessed by graphical analysis (goodness-of-fit plots) and pcVPC. Cross-validation or external validation was not used due to the limitation of sample sizes (Colby and Bair, 2013). The goodness-of-fit diagnostic plots are shown in Figure 1. The SOFA score of some subjects did not decrease, indicating that these subjects had no response to the standard care therapy and kukoamine B. Most dots of CWRES were shown to lie within an acceptable range (−2 to 2), which suggested little to no bias over time. Most dots of absolute value of IWRES lay within an acceptable range (0–2), indicating no bias with the predicted value. pcVPC stratified on dose was carried out with 1,000 simulations, the plot with 80% confidence interval (low sample size) is shown in Figure 2. Most of the observed SOFA scores were within the 80% CI of the model-predicted SOFA score, demonstrating adequate model prediction. In the 0.06 mg/kg group and 0.12 mg/kg group, the 10th and 90th percentiles of the predicted values were wider than the 10th and 90th percentiles of the observed values. It was elucidated that the random effect predicted was higher than the observed values because of a low sample size. The 50th percentiles of the observed values and predicted values were generally consistent. In the placebo group, the standard care effect was somewhat overestimated, which was assumed to be related to the high heterogeneity of sepsis patients.

### Simulation

**FIGURE 3:**
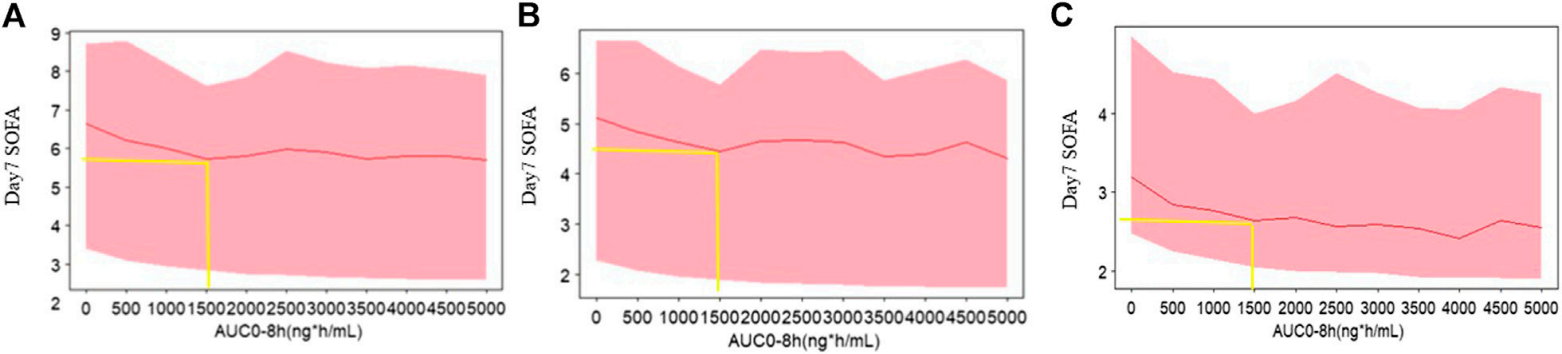
The simulated profiles of day 7 SOFA vs. AUC based on the exposure model **(A)** baseline 15, **(B)** baseline 10, and **(C)** baseline 5. The red lines represent the 50th percentiles of SOFA score distribution in each panel. The upper and lower bound of shadow represent the 30th and 70th percentiles of SOFA score distribution in each panel.

**FIGURE 4:**
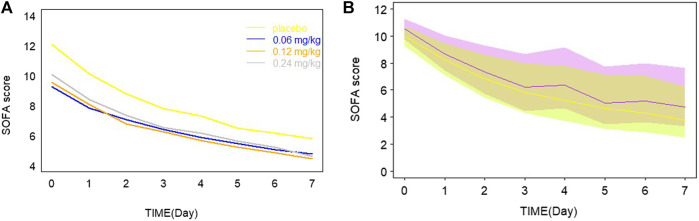
**(A)** Typical model predictions plot overlaying the effect of placebo and different dosage. **(B)** The simulated profiles of SOFA score vs. time in the recommended 0.24 mg/kg drug groups and the placebo group in a panel. The purple line and shadow bounds represent the 30th, 50th, and 70th percentiles of SOFA score distribution in the placebo group. The yellow line and shadow bounds represent the 30th, 50th, and 70th percentiles of SOFA score distribution in the 0.24 mg/kg drug groups.

Day 7 SOFA vs. AUC profiles were simulated, which are shown in Figure 3. It was observed that day 7 SOFA decreased to a plateau when AUC was 1,500 h* ng/mL and the patients were administrated for 1 h an intravenous infusion of kukoamine B every 8 h for seven continuous days. Furthermore, SOFA score decreased more significantly when the baseline SOFA was high. It provided some important information for the inclusion criteria of subjects in the following Phase IIb clinical trial. Besides, SOFA score vs. time profiles were also simulated, and the plots overlaying the effect of the placebo and the recommend 0.24 mg/kg dosing regimen are shown in Figure 4. A difference in the efficacy of the placebo group and the 0.24 mg/kg group was observed. In the 0.24 mg/kg dosing regimen, no serious adverse effect was investigated in the Phase IIa trial. For 80% of patients, AUC can achieve 1,500 h*ng/mL. Based on the simulated results and good safety, we recommended the 0.24 mg/kg dosing regimen for the Phase IIb clinical trial.

## Discussion

Sepsis, irregulated systemic inflammatory response, is triggered by severe infection. Confounding factors such as heterogeneous clinical presentation, various causative pathogens, and different sites of infection, all contribute to disease progression. SOFA score is widely used in ICUs to evaluate disease severity and predict sepsis patients’ outcome. Patients’ baseline SOFA scores in our study had a wide range, representing all possible characteristics of this disease. The change trend of patients’ SOFA score also had a large difference.

Exposure-response (E-R) relationship was important for optimizing dose selection in the drug development. PK measure in the E-R relationship should be properly selected based on the mechanism of action of kukoamine B. Considering that AUC is often more accurately estimated, AUCs were thought to be related to the pharmacological effect from the perspective of the mechanism of action of kukoamine B in our study. Thus, AUC was finally selected as the PK measure to build the E-R model (Krzyzanski and Jusko, 1998) The drug effect R (t) was a latent variable which was described by the IDR model consisting of a zero-order increase of R (t) and a one-order decrease of R (t). Because kukoamine B exerts anti-inflammatory effects based on its potent affinity with LPS and CpG DNA, drug effect in zero-order increased the SOFA score. The mixture model was tested considering that some subjects had no response to kukoamine B and the standard care therapy. However, the OFV minimization failed. It was thought that the number of subjects with no response was low. The observed baseline SOFA score as a variable was included in the model. In the primary stage of model development, the baseline was estimated as a parameter, but the variability of the baseline was high (487.1%). Sources of the variability cannot be explained, so the observed baseline SOFA as a variable was directly included in the model. In our study, for patients’ safety, a total of eight patients with mild sepsis were firstly randomized at a 3:1 ratio to the 0.06 mg/kg drug group and the placebo group. The baseline SOFA was the lowest in the 0.06 mg/kg dose group. In the formal trial, the total of 36 patients were randomized at a 1:1:1 ratio into the 0.12 mg/kg, 0.24 mg/kg, and placebo groups. The group difference of the baseline SOFA was attributed to the low sample size.

In the model building step, several structural models based on the graphic exploration and prior knowledge were explored. The results are summarized in Supplementary Table S2. This model structure was not applied because of the failed model minimization or high RSE% of the parameters or some model shortcomings. The final model had some limits as follows: 1) some parameters were fixed to achieve successful minimization; 2) pcVPC results indicated that random variability may be overestimated especially for the 0.06 mg/kg group and 0.12 mg/kg group. It was elucidated that SOFA score had a high interindividual and intraindividual variability (shown in Supplementary Figure S2) and the sample size was low. 3) pcVPC plot indicated that the standard care effect was somewhat overestimated for the placebo group. It was assumed to be related to the limited sample size and the high heterogeneity of sepsis patients. The model would be validated by large population data in the following Phase IIb clinical trial.

From model estimation results, the maximum fraction of the placebo effect was estimated to be 0.792, while the maximum fraction of the drug effect was 0.208. The drug effect was modest compared with the standard care therapy effect. From Figure 4, a small difference between groups was observed. These results may be limited by the low sample size, but would be validated by the following Phase IIb data. Day 7 SOFA was simulated when AUC of kukoamine B ranged from 0 to 5,000 h*ng/mL, based on the built model. When AUC was 1,500 ng/ml*h, day 7 SOFA decreased to a plateau. The pharmacokinetic study of kukoamine B in sepsis patients showed that the average AUC_0–8 h_ of the 0.24 mg/kg group patients was 1,500 h*ng/mL. Thus, the 0.24 mg/kg dosing regimen was recommended for the Phase IIb clinical trial.

Up to date, no report about an exposure-response model using the SOFA score has been published (Hu et al., 2014; Hu et al., 2016). In our study, the exposure-response model linking AUC and SOFA score incorporating the standard care effect and the drug effect was developed for the first time. The built model can not only be used for dosing optimization for kukoamine B in the following clinical trials, but could also be utilized in the clinical trials of similar drugs in which SOFA score is used as a biomarker of efficacy. Our study had some limitations like the low sample size and no identified influence factors. In the future, important covariates could be identified in a large population.

## Data Availability

The raw data supporting the conclusions of this article will be made available by the authors, without undue reservation.
