# Supplementary material for: Exposure-Response Modeling to Support Dosing Selection for Phase IIb Development of Kukoamine B in Sepsis Patients
Source: Front Pharmacol. 2021 Apr 19;12:645130. doi: 10.3389/fphar.2021.645130 (PMC8091127; doi:10.3389/fphar.2021.645130)
Supplement: Supplementary file 1 [file datasheet1.docx]

**Table S1 Numbers of Subjects and Observations for the Final Exposure-response Model Dataset**

| Treatment | Number of Subjects | | Number of PK samples | Number of SOFA observations |
| --- | --- | --- | --- | --- |
| Placebo* | 10 | 0 | | 71 |
| 0.06 mg/kg | 4 | 76 | | 32 |
| 0.12 mg/kg | 9 | 121 | | 61 |
| 0.24 mg/kg | 11 | 131 | | 81 |
| Total | 34 | 328 | | 245 |

Placebo*: The patients administrated for the standard care therapy.

**Table S2 Summary of structural models**

| Key models | Model structures | Objective function value | Comments |
| --- | --- | --- | --- |
| Exponential progression model with an addition function | R(t)=R0$\times$exp(-k1$\times$t -k2$\times$AUC$\times$t) | 639.88 | OFV minimization successful, the RSE% of the parameters was 176%. |
| Exponential progression model with an power function | R(t)=R0$\times$exp(-k$\times$AUC^^power^$\times$t) | 490.15 | OFV minimization terminated  due to rounding errors |
| Maximum effect model with accumulation AUC | $E=E_{base}\times e^{-kt}-(E_{base}-E_{max}\times\frac{{AUC}^{r}}{{AUC}^{r}+{{EAUC}_{50}}^{r}}$ | 603.97 | The model can well describe the observed data, but accumulated AUC was used in the model. The model can’t describe the progression process of the disease |
| sequential model  approach with the standard care effect and drug effect | $SOFA\left( t \right)=Base-Base\times\left( 1-e^{-kt} \right)+\varepsilon$  $SOFA\left( t \right)=Base-Base\times\left( 1-e^{-kt} \right)-Base\times\left( 1-R\left( t \right) \right)+\varepsilon$  $\frac{dR(t)}{dt}=k_{in}\times(1-\frac{AUC}{AUC+E{AUC}_{50}})-k_{out}\times R(t)$ | 487.77 | (1) The proposed model structure can provide negative values if complete R inhibition and long times  (2) The RSE% of EAUC_50_ was high. |
| A simultaneous model approach with the standard care effect and drug effect(linear) | $SOFA\left( t \right)=Base-F_{placebo}\times Base\times\left( 1-e^{-kt} \right)-\left( 1-F_{placebo} \right)\times Base\times\left( 1-R\left( t \right) \right)+\varepsilon$  $\frac{dR(t)}{dt}=k_{in}\times(1-b\times AUC)-k_{out}\times R(t)$ | 589.48 | Covariance step aborted |
