# Supplementary material for: Exposure-Response Modeling to Support Dosing Selection for Phase IIb Development of Kukoamine B in Sepsis Patients
Source: Front Pharmacol. 2021 Apr 19;12:645130. doi: 10.3389/fphar.2021.645130 (PMC8091127; doi:10.3389/fphar.2021.645130)
Supplement: Supplementary file 2 [file presentation1.pptx]

## Slide 1
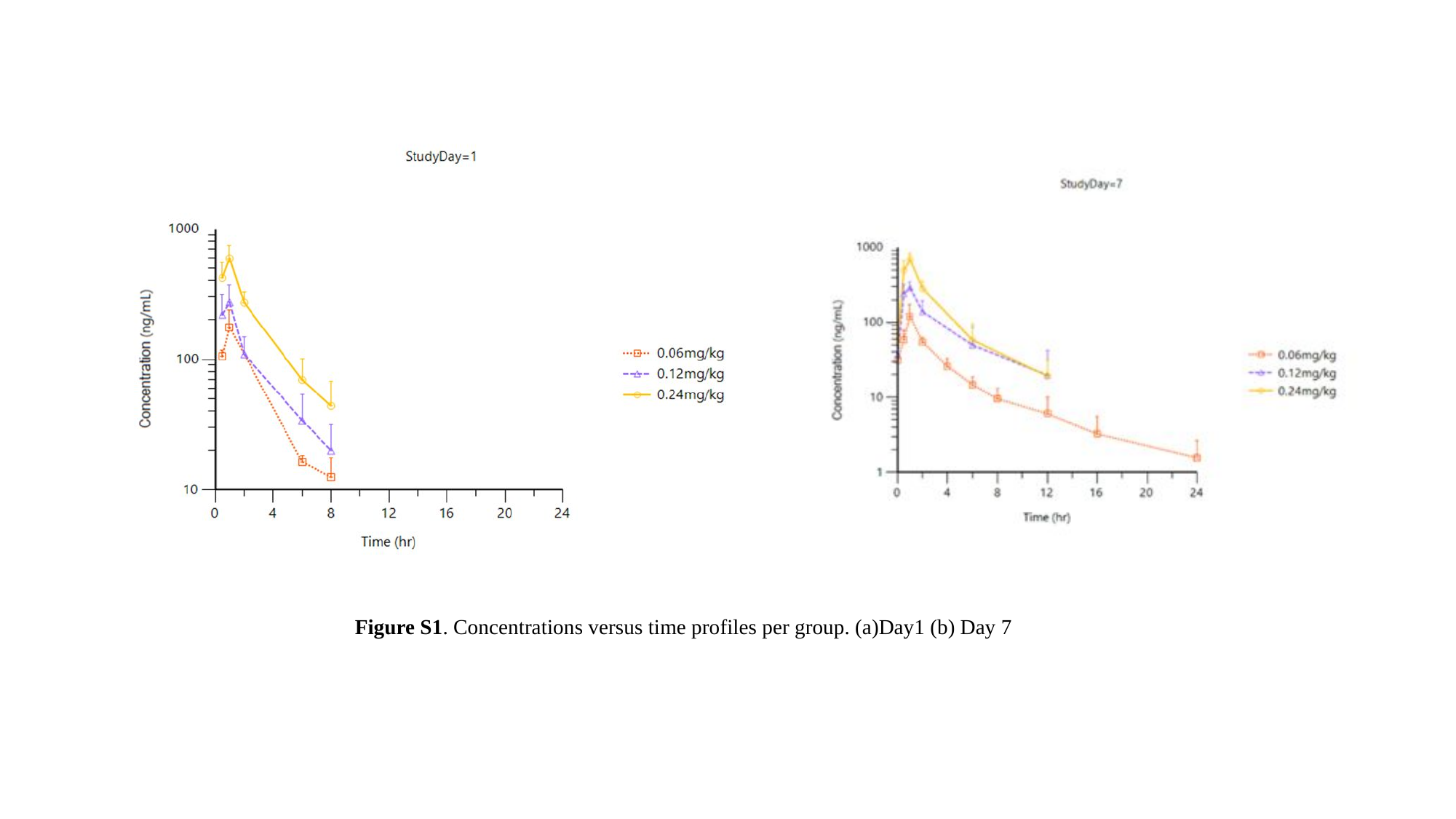

Figure S1. Concentrations versus time profiles per group. (a)Day1 (b) Day 7

## Slide 2
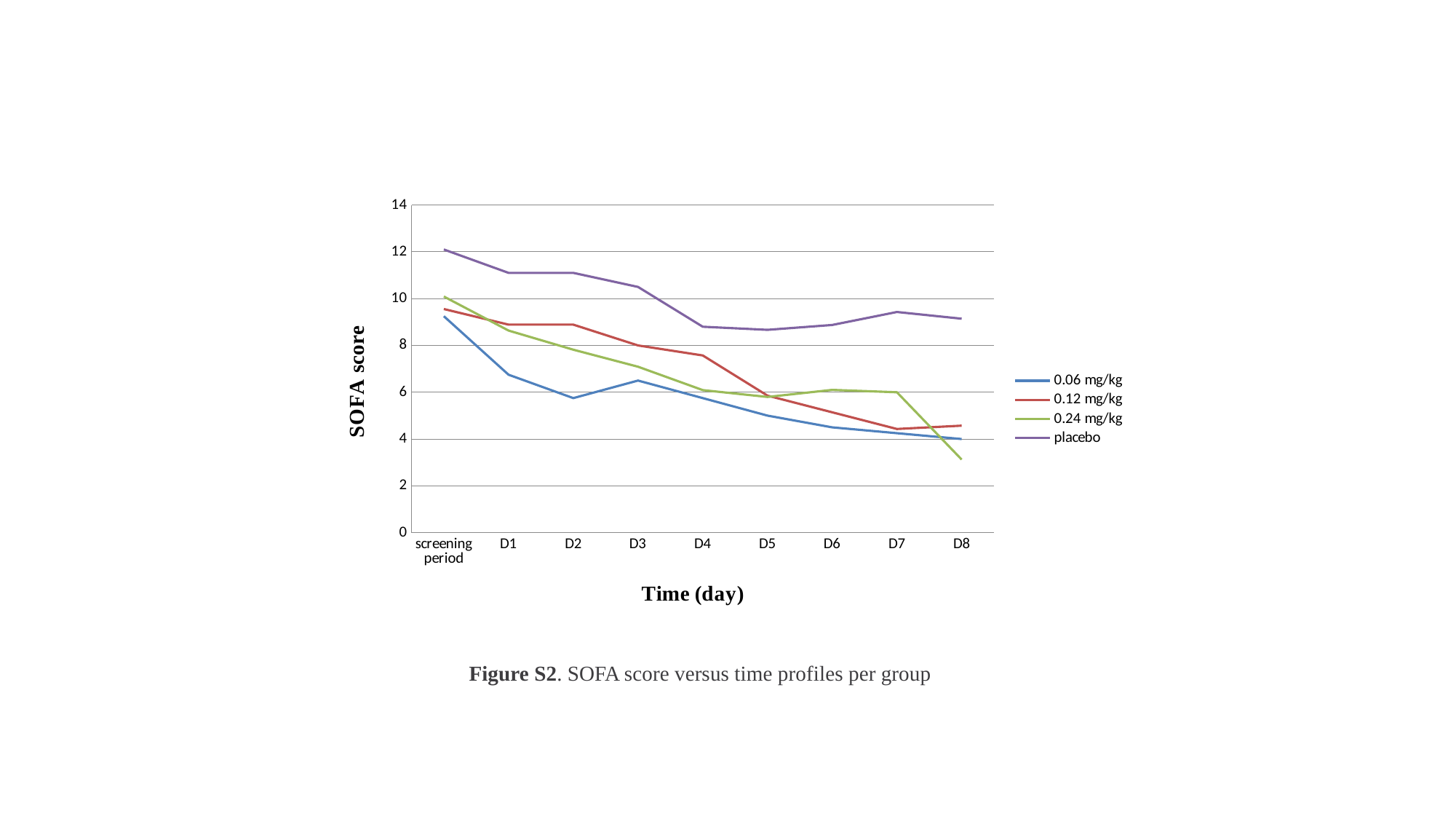

### Chart
| Category | | | | |
|---|---|---|---|---|
| screening period | 9.25 | 9.555555555555555 | 10.090909090909092 | 12.1 |
| D1 | 6.75 | 8.88888888888889 | 8.636363636363637 | 11.1 |
| D2 | 5.75 | 8.88888888888889 | 7.818181818181818 | 11.1 |
| D3 | 6.5 | 8.0 | 7.090909090909091 | 10.5 |
| D4 | 5.75 | 7.571428571428571 | 6.090909090909091 | 8.8 |
| D5 | 5.0 | 5.857142857142857 | 5.8 | 8.666666666666666 |
| D6 | 4.5 | 5.142857142857143 | 6.1 | 8.875 |
| D7 | 4.25 | 4.428571428571429 | 6.0 | 9.428571428571429 |
| D8 | 4.0 | 4.571428571428571 | 3.125 | 9.142857142857142 |Figure S2. SOFA score versus time profiles per group
